# Impact of the COVID-19 pandemic on the digitization of routine pediatric practice in Spain: A nationwide survey study

**DOI:** 10.3389/fped.2023.1106488

**Published:** 2023-02-14

**Authors:** Rafael Martín-Masot, Juan J. Diaz-Martin, Alicia Santamaría-Orleans, Víctor Manuel Navas-López

**Affiliations:** ^1^ Pediatric Gastroenterology and Nutrition Unit, Hospital Regional Universitario de Malaga, Málaga, Spain; ^2^ Pediatric Gastroenterology and Nutrition Unit, Hospital Universitario Central de Asturias, Oviedo, Spain; ^3^ Department of Scientific Communication, Laboratorios Ordesa S.L., Barcelona, Spain

**Keywords:** pediatrics, COVID-19, pediatric practice, telemedicine, digitization, infant feeding

## Abstract

**Introduction:**

The COVID-19 pandemic forced a change in the working dynamics of all healthcare professionals, leading to the sudden introduction of telemedicine. Although until that moment telemedicine applications had been described in the paediatric age, their use was anecdotal.

**Objective:**

To analyse the experience of Spanish paediatricians after the forced digitization of consultations due to the pandemic.

**Methods:**

A cross-sectional survey-type study was designed to obtain information from Spanish paediatricians about the changes that took place in the usual clinical practice.

**Results:**

306 health professionals participated in the study Most of them agreed on the use of the internet and social networks during the pandemic, referring to mail or WhatsApp® as usual channels of communication with their patients' families. There was a great agreement among paediatricians that the evaluation of newborns after hospital discharge and establishing methodologies that allow childhood vaccination and the identification of subsidiary patients for face-to-face evaluation were necessary although the limitations of the lockdown. The idea that telephone and digital consultations have optimized the consultation time and that they will probably continue after the end of the pandemic was generally accepted. No changes in adherence to breastfeeding or the start of complementary feeding were referred to, but an increase in the duration of breastfeeding and the appearance of frequent hoaxes in social networks concerning infant feeding were found.

**Conclusions:**

It is necessary to analyse the impact of telemedicine in paediatric consultations during the pandemic to evaluate its effectiveness and quality to maintain it in routine paediatric practice.

## Introduction

In December 2019, the outbreak of an infection by a new coronavirus (SARS-CoV-2) detected in Wuhan (China) began (1). Subsequently, the World Health Organization (WHO) made an international emergency declaration, which resulted in population confinements in most countries in the following months. In Spain, in March 2020, the state of alarm was decreed, forcing the work dynamics of all medical professionals to be modified, leading to a labor reorganization, implementation of virtual consultations, electronic medical consultations, and telemedicine. In addition to limiting access to the hospital environment, confinement decreased adherence to treatment and the number of scheduled interventions (2).

Until then, telemedicine was booming, especially in Hospital Care and in adult patients, mainly in some specialties such as Dermatology (3). It is a tool that can respond to health problems with a shorter response time than the usual channels, allowing the patient to benefit from care without having to travel (4), an essential situation once population confinement has been declared.

Although the applications of telemedicine were previously described for the paediatric population, the COVID-19 pandemic precipitated its establishment without prior preparation, and with reduced prior experience (5). While the advantages of the correct use of telemedicine are promising (6), it is necessary to analyse the experience of paediatricians on its establishment, advantages, disadvantages, and facilitating elements for its future implementation. Previous studies (7, 8) have reported barriers to its use in paediatric age, such as limitations for diagnostic tests or impossibility of physical examination. The pandemic has reduced throat cultures, and screening programs, including delayed screening for neonatal metabolic disorders, feeding consultations, or rehabilitation and physiotherapy programs. However, it is necessary to analyse the circumstances of each environment about its health system and its population.

The fact that it has been implemented at even more vulnerable ages during the pandemic, both psychologically and emotionally (9) as well as critical for growth and neurological development, makes it essential to recognize potential problems with its establishment at this age. In a world in which remote care is here to stay, it is necessary to discuss the current healthcare model, and to know what measures could be established in the paediatric routine to improve patient care in case of future needs. Therefore, the objective of the study was to collect information on the changes that paediatricians made in Spain due to the COVID-19 pandemic, and how it may have influenced infant feeding practices and in general paediatric care.

## Materials and methods

### Design

A cross-sectional survey study was designed to gather information from specialists in Paediatrics working in the outpatient setting throughout Spain. For the study, a team of professionals in the health care and sociology fields with experience in survey studies developed the questionnaire. The survey consisted of 32 questions. Open questions were included for sociodemographic variables (7 questions). The survey was divided into two blocks: digitization of the paediatric consultation and social networks (10 and 9 questions respectively) and impact on feeding patterns (6 questions). The answers about changes in regular medical consultation were based on the degree of agreement on the question asked, acquiring values from 1 if “totally disagree” to 5 if “totally agree”. For two questions about the impact of the pandemic on feeding patterns, a questionnaire was carried out on 10 points instead of 5 points (10 maximum agreement; 1 maximum disagreement). The survey was conducted from March to October 2021. The sample was non-randomized and proportionally stratified to the number of specialists in paediatrics registered in the autonomous communities of the country.

### Subjects

Paediatricians from the public and private sectors, hospital care, and primary care were included. Candidates to participate in the study were specialists in paediatrics involved in the care of children who attended public or private consultations throughout Spain. The participants were recruited through invitations to specialists in paediatrics registered in the database of Ordesa Laboratories of more than 5,000 specialists, a pharmaceutical company specialized in infant nutrition and paediatric food supplements. Participation in the study was anonymous and voluntary. Paediatricians who met the inclusion criteria (more than 5 years of experience in paediatric consultation and have been professionally active during the last 12 months) and accepted to participate in the study were provided with the data collection logbook.

### Data analyses

Descriptive statistics (mean, standard deviation) for quantitative variables and percentage of participants (%) for categorical variables to describe the baseline characteristics of the study sample were employed. Fisher's exact test to explore the association between qualitative variables was conducted. Statistical analyses were performed using IBM SPSS Statistics version 23.0 (IBM, Armonk, NY, USA). *p* < 0.05 was considered significant.

## Results

### Sociodemographic data

306 surveys (5,6% of response percentage) were collected. 52% of the participants in the present study were women. Participants had a mean age of 54 years and an average of 27 years of exercise experience. 84% of those interviewed worked in an urban environment. Data are shown in Table 1.

**Table 1:** Sociodemographic characteristics of the study participants.

Variable	Paediatricians (*n* = 306)
Age (years)	54.2 (9.6)
Sex (female)	160 (52)
Experience (years)	26.8 (9.9)
**Nationality**	
* Spanish*	291 (95)
* Latin American*	9 (3)
* Other countries of the European Union*	6 (2)
**Environment**	
* Urban*	257 (84)
* Rural/semi-urban*	29 (9.5)
**Area**	
* Public sector*	116 (38)
* Private sector*	155 (50.7)
* Both*	34 (11)
**Type of institution**	
* Hospital care*	87 (28.4)
* Primary care*	170 (55.6)

Results are expressed as number (%) or mean (standard deviation) unless otherwise indicated.

### Digitization of pediatric consultation and social networks

93% of the participants consider the internet to be a fairly useful or very useful source of information, and 86% of those interviewed mention that they normally use digital tools as a communication channel with their patients’ families, being the more frequently used mail (87%) and WhatsApp® (52%). Statistically significant differences have been detected by area (*p* = 0.008), with the percentage of use of WhatsApp®, blog, phone call, video consultation, and telemedicine platforms being higher in private medicine.

**Figure 1:**
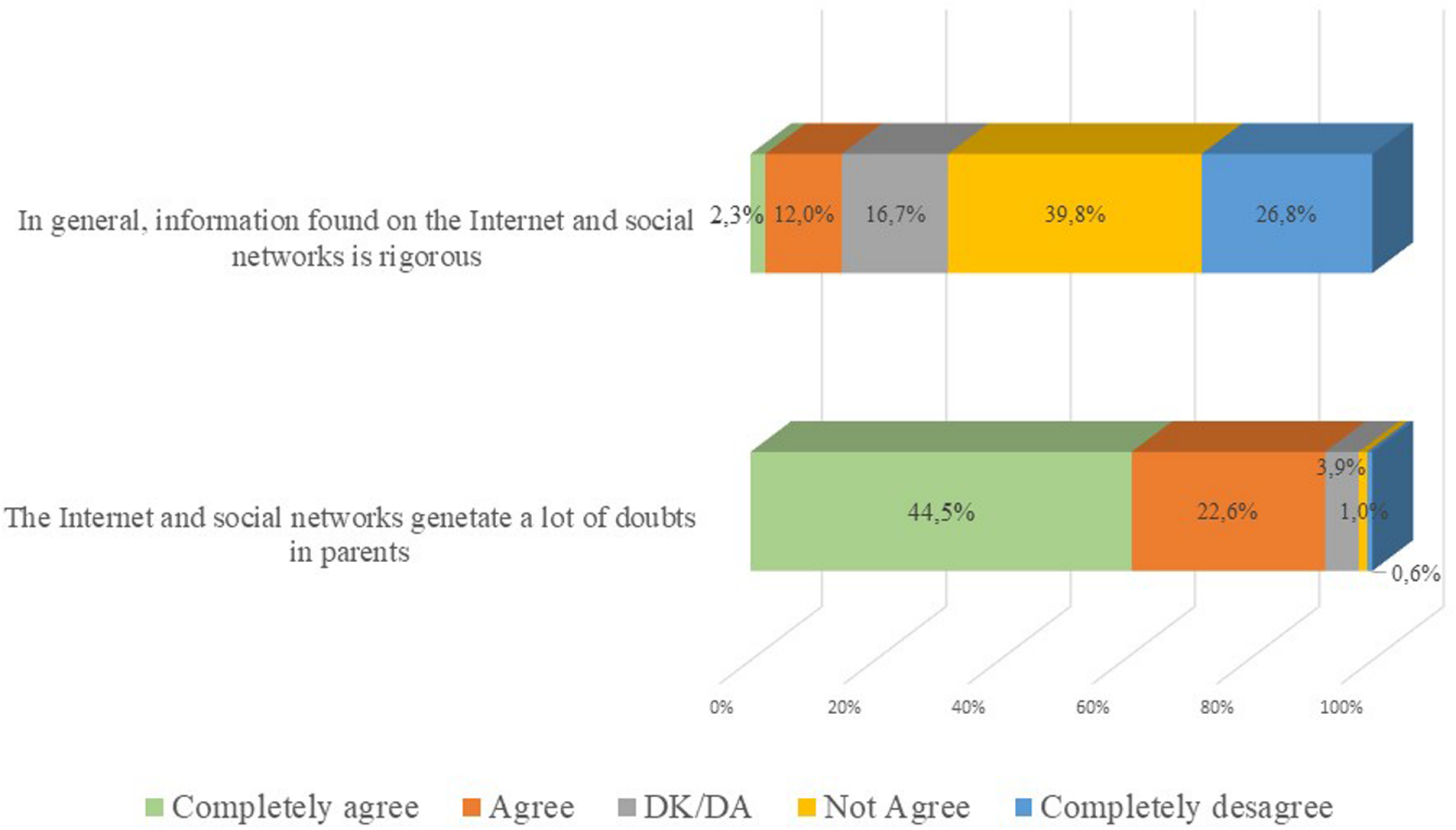
Statements on the internet and social networks as sources of information.

For the statements: “the internet and social networks generate many doubts in parents” and “Information found on the Internet and social networks is rigorous”, a questionnaire was carried out on 10 points (10 maximum agreement; 1 maximum disagreement), obtaining an average of 8.24 in the first vs. 3.75 in the second. The percentages are shown in Figure 1.

Table 2 shows the statements with the highest percentage of agreement among the paediatricians surveyed, and the differences observed according to sociodemographic characteristics. As shown, in general, there was a greater discrepancy depending on the area and the type of institution.

**Table 2:** Statements with the highest degree of agreement among the paediatricians surveyed.

Statements	Degree of agreement (mean)*	Sex (*p*-value)	Age (*p*-value)	Environment (*p*-value)	Area (*p*-value)	Type of institution (*p*-value)
All newborns should be seen by a paediatrician in person shortly after hospital discharge	*4* *.* *74*	0.41	***<0*** ***.*** ***01***	0.19	0.18	0.55
It is essential to establish a methodology that allows easy identification of children who have missed visits to the Child Health Program and recommended vaccinations.	*4* *.* *54*	0.75	0.15	0.39	0.87	0.81
It is advisable to consider a strategy on what are the objectives and messages that you want to transmit through digital communications	*4* *.* *35*	0.36	0.9	0.3	0.81	0.89
Many healthcare and especially bureaucratic queries have been resolved remotely, avoiding travel	*4.27*	0.41	0.41	0.5	***<0*** ***.*** ***01***	***<0*** ***.*** ***01***
Incorporation of telephone and digital consultations has been useful for monitoring children suspected of having SARS-CoV-2	*4.27*	0.74	0.45	0.9	***<0*** ***.*** ***01***	***<0*** ***.*** ***01***
Telephone and digital consultations have proven to be extremely useful in the triage of patients	*4.11*	***0*** ***.*** ***02***	0.82	0.3	***<0*** ***.*** ***01***	***<0*** ***.*** ***01***
Telephone and digital consultations would also be useful in a COVID-free period to optimize time availability.	*4.07*	0.08	0.35	0.69	***<0*** ***.*** ***01***	***<0*** ***.*** ***01***
Telephone and digital consultations will continue in the paediatric consultation once the current pandemic situation ends	*3* *.* *99*	***<0*** ***.*** ***01***	0.06	0.36	0.16	***0*** ***.*** ***04***
Digital tools allow doctor-patient interactivity beyond the consultation and provide added value	*3* *.* *87*	0.95	0.92	0.9	0.28	0.87

Results of Fisher's exact test are shown.

* Values ranking from 1 if “totally disagree” to 5 if “totally agree”.

Bold and italic values represent the *p* values <0.05.

### Impact of the pandemic on infant feeding patterns

No differences were detected in the prevalence of breastfeeding (40% of paediatricians detected an increase in breastfeeding vs. 54% who did not consider it), although a longer duration of breastfeeding due to the pandemic was observed (66%).

The vast majority of those surveyed (88%) do not consider that the economic crisis caused by COVID has caused an early introduction of complementary feeding.

85% of the participants report that parents ask about the information they have found on social networks. Doubts related to infant feeding were the most frequently reported hoaxes. In fact, up to 49% of those surveyed detected that caregivers were influenced by hoaxes related to COVID-19 collected on social networks and the internet, with significant differences by area and type of institution (higher in urban areas, *p* = 0.04; and in-hospital care, *p* = 0.02) with up to 32.5% of these hoaxes being about influence of COVID-19 on breastfeeding (ahead of vaccines, 23%) (Table 3).

**Table 3:** Caregivers influenced by hoaxes (*n* = 151).

Breastfeeding	49 (32.5)
Vaccine	35 (23.2)
Contagion/transmission	26 (17.2)
Feeding	6 (4)
Others	47 (31)

Values shown as number (%) of total hoaxes.

For requirements for a specific article type please refer to the Article Types on any Frontiers journal page. Please also refer to Author Guidelines for further information on how to organize your manuscript in the required sections or their equivalents for your field.^1^

## Discussion

The present article expounds the first analysis of the impact of the COVID-19 pandemic on the routine practice of paediatricians in Spain. It was observed that most of the paediatricians surveyed reported the use of digital communications with their patients’ families, although limited face-to-face patient assessment was an option during the study period.

Digitization of paediatric consultation is an increasingly frequent alternative. Previous own data (unpublished) showed that 51.5% in 2017 (NUTRILAC study) and 49.8% in 2019 (OPTILAC study) used digital communication with their patients in Spain. In our data from 2021, after the state of alarm and confinement due to the pandemic in our environment, this percentage has increased to 86% and shows a tendency already described by other authors (10).

Regarding the limitations of digital communications between paediatricians and patients, data shown by the European Paediatric Association, Union of National European Paediatric Societies and Associations (EPA-UNEPSA) recently indicated a marked reduction in face-to-face patient care, also showing that in some European countries, paediatricians have decreased the performance of pharyngeal cultures and diagnostic tests in general, as well as primary prevention services, such as child health programs, neonatal screening or consultations on dietary habits (8). Telemedicine has been attributed to diagnostic delays, decreased care for chronic patients (9), and medical overtreatment due to the inability to develop a proper assessment (8).

Some health consultations are not easily solved in digital practice. In our study, Spanish paediatricians were generally agreeing whit the idea that it is necessary to examine newborns a few days after delivery. Likewise, there was a great agreement that it is necessary to establish a methodology that allows the detection of subsidiary patients for physical assessment and avoids preventing controls and regular vaccines from the Child Health Program from being lost.

Another limitation related to telemedicine is the need to supply health services with the necessary tools for telematic care (11). In addition, it should be necessary that there was a legal regulation of health care through telemedicine, with the establishment of protocols for the virtual assessment of patients, due to its ethical and legal implications. Its abrupt establishment has prevented they could be developed in parallel.

However, telemedicine presents many advantages, such as the possibility of attending to patients without the need to travel, reduction in response times, reduction of costs, or reduction of the risk of spreading infections. Studies carried out during the pandemic in different settings have shown a high patient’s satisfaction with this new methodology (12–14) and that it can even help to improve control in some chronic diseases (15). In addition, the establishment of telemedicine has allowed various problems to be solved remotely, avoiding unnecessary travel, and “bringing” health centres closer to patients. In our study, paediatricians agree that telephone and digital consultations optimize time availability.

Probably, the creation of mixed agendas (with telematic and face-to-face assessment), as patients themselves suggested in some studies (16), could contribute to more efficiency in the system. In the same way, it can contribute to reducing school and work absenteeism, as has been shown in studies carried out during the pandemic (17), and can also be considered as a cost-effective strategy. In fact, the proactive use of telemedicine by the health system has been described as much more beneficial than the reactive one (18), which has led many countries to consider its implementation in routine practice (19).

The evolution of medicine and technology favours that patients can obtain information about their health status more efficiently than previously. Social networks are useful to improve patient care, connect them with health professionals, reach a larger population and reduce consultation time (20). However, in our study, most of the paediatricians surveyed warned about the danger that not professional reviewed digital sources may entail, strongly agreeing with the statement that the information obtained by patients *via* the Internet is probably little rigorous. Data obtained in the survey on infant feeding also warn about this problem. Paediatricians agreed that parents asked them about hoaxes and misinformation that have been found through social networks, especially concerning breastfeeding. In general, health and scientific subjects have been identified as the hoaxes most frequently detected during the pandemic in our country, with social networks being the most used platforms for their dissemination (21). This fact could be partially solved if paediatricians establish educational measures on reliable sources of information, providing trusted Internet portals to patients (22).

Regarding changes in infants eating patterns, several studies recently published (23–25) had coincided with a worsening of eating habits during the pandemic, especially in lower social strata, which could increase the future figures of obesity. Likewise, some authors have reported a decrease in breastfeeding rates as a result of COVID-19, due to various factors (26, 27). Our data, however, support the idea that although an increase in breastfeeding was not detected, confinement did lead to a longer duration of breastfeeding. One of the causes could be the impossibility of mothers joining work and the increase of time cohabitation with infants, being telemedicine able to help breastfeeding mothers in this situation (28). The fact that a longer duration of breastfeeding has been related to lower rates of obesity in adulthood (29, 30), probably makes it necessary to debate not only the time of maternity leave in our environment, in the public and private sectors, but also the idea that teleworking can be an easy tool for facilitating its increasing. In our survey, paediatricians did not detected an early introduction of complementary feeding, a situation that in healthy infants under 6 months does not seem to provide any benefit in developed countries (31).

Although our study has several limitations, such as the lack of representativity of the general Spanish paediatrician’s profile, its strengths must the considered. It is the first study on real practice carried out by Spanish paediatricians during the pandemic period and includes a large number of participants. In any case, the data obtained must be corroborated after the end of the pandemic, to assess the real impact of the pandemic on normal practice. In addition, to complement the results, it would be necessary to analyse patients’ satisfaction with the care offered during this period.

Based on our data, it can be concluded that consultation digitization was widely implemented during the pandemic period in Spanish paediatric consultations and its benefits were positively considered by paediatricians. Telemedicine is a present reality and probably complementary to classic paediatric care, being a tool that is highly valued by paediatricians in Spain. Although there is room for improvement, the establishment of protocols and the adaptation of telemedicine applications to the paediatric age population will probably contribute to more efficient and quality care for our patients.

## Data Availability

The raw data supporting the conclusions of this article will be made available by the authors, without undue reservation.

## Footnotes

^1^For Original Research articles, please note that the Material and Methods section can be placed in any of the following ways: before Results, before Discussion or after Discussion.
